# Compact Quantum Cascade Laser-Based Noninvasive Glucose Sensor Upgraded with Direct Comb Data-Mining

**DOI:** 10.3390/s25020587

**Published:** 2025-01-20

**Authors:** Liying Song, Zhiqiang Han, Hengyong Nie, Woon-Ming Lau

**Affiliations:** 1 School of Mathematics and Physics, University of Science and Technology Beijing, Beijing 100083, China; songliying.sly@foxmail.com; 2 Shunde Innovation School, University of Science and Technology Beijing, Foshan 528399, China; 3 School of Chemistry and Chemical Engineering, Linyi University, Linyi 276000, China; hnie@uwo.ca; 4 The Sun Age New Energy Ltd., Zhuhai 519100, China; 5 The Yaoling Age New Energy Technology Development Ltd., Linyi 276000, China; 6 Surface Science Western, Western University, London, ON N6G 0J3, Canada; 7 Department of Physics and Astronomy, Western University, London, ON N6A 3K7, Canada

**Keywords:** compact mid-infrared sensor, data-mining, noninvasive glucose, quantum cascade laser

## Abstract

Mid-infrared spectral analysis has long been recognized as the most accurate noninvasive blood glucose measurement method, yet no practical compact mid-infrared blood glucose sensor has ever passed the accuracy benchmark set by the USA Food and Drug Administration (FDA): to substitute for the finger-pricking glucometers in the market, a new sensor must first show that 95% of their glucose measurements have errors below 15% of these glucometers. Although recent innovative exploitations of the well-established Fourier-transform infrared (FTIR) spectroscopy have reached such FDA accuracy benchmarks, an FTIR spectrometer is too bulky. The advancements of quantum cascade lasers (QCLs) can lead to FTIR spectrometers of reduced size, but compact QCL-based noninvasive blood glucose sensors are not yet available. This work reports on two compact sensor system designs, both reaching the FDA accuracy benchmark. Each design commonly comprises a mid-infrared QCL for emission, a multiple attenuation total reflection prism (MATR) for data acquisition, and a computer-controlled infrared detector for data analysis. The first design translates the comb-like signals into conventional spectra, and then data-mines the resultant spectra to yield blood glucose concentrations. When a pressure actuator is employed to press the patient’s hypothenar against the MATR, the sensor accuracy is considered to reach the FDA accuracy benchmark. The second design abandons the data processing step of translating combs-to-spectra and directly data-mines the “first-hand” comb signal. Beyond increasing the measurement accuracy to the FDA accuracy benchmark, even without a pressure actuator, direct comb data-mining upgrades the sensor system with speed and data integrity, which can impact the healthcare of diabetic patients. Specifically, the sensor performance is validated with 492 glucose absorption scans in the time domain, each with 20 million datapoints measured from four subjects with glucose concentrations of 3.9–7.9 mM. The sensor data-mines 164 sets of critical singularity strengths, each comprising 4 critical singularity strengths directly from the 9840 million raw signal datapoints, and the 656 critical singularity strengths are subjected to a machine-learning regression model analysis, which yields 164 glucose concentrations. These concentrations are correlated with those measured with a standard finger-pricking glucometer. An accuracy of 99.6% is confirmed from the 164 measurements with errors not more than 15% from the reference of the standard glucometer.

## 1. Introduction

The demand for innovative blood glucose sensors is high because, presently, over 500 million people are suffering from diabetes, who are advised to monitor their blood glucose levels on a daily basis. Furthermore, the population with diabetes is rising and the starting age of hyperglycemia is persistently dropping, with a present threshold of 35 [1]. Although diabetes cannot be healed, tracking blood glucose concentrations facilitates its adequate management and reduces the risks of fatal health complications. The most recent statistics [2] disclosed by the World Health Organization show that noncommunicable diseases kill 41 million people each year, equivalent to 74% of all deaths globally, and the four most dangerous diseases are cardiovascular diseases (17.9 million deaths), cancers (9.3 million), chronic respiratory diseases (4.1 million), and diabetes (2.0 million). Advancements in the healthcare of diabetes urgently need innovative blood glucose sensors.

Prevalently, blood glucose tests are conducted with hand-held glucometers which detect the electrical conductivity on a glucose test strip. In each test, a patient places a drop of blood from finger pricking onto a test strip where the blood’s glucose is oxidized by a preloaded glucose specific enzyme to form electrically conductive gluconic acid. Although an array of micro-needles strapped tightly to a patient is now commercially available to ease the inconvenient and slow finger-pricking process, the invasive nature and the associative health risks associated with this approach remain [3,4,5]. In principle, spectroscopic detection of the molecular signatures of glucose in the interstitial fluid via light penetration to and from the subcutaneous microcirculation network of a patient can be noninvasive and fast [6,7,8].

**Figure 1:**
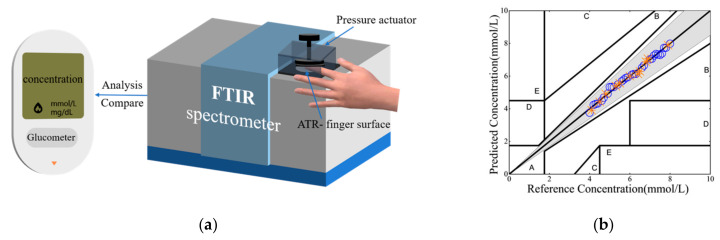
(**a**) Schematic of an FTIR-based sensor equipped with a single-pass ATR plus a pressure actuator [17]. (**b**) The Clarke error grid plot for such a sensor with spectral analysis of the region of 1000 to 1040 cm^−1^ for reducing spectral interference, with data collection from the hypothenar replacing that from the finger [18] (the blue circles are the data of the training set, and the orange crosses are the data of the testing set).

Although the research and development of noninvasive blood glucose sensors by infrared, Raman, and photoacoustic spectroscopies have been active for decades [9,10,11,12,13,14,15,16], the intensive global effort has yielded limited successes. For example, the USA Food and Drug Administration (FDA) published the directory “Self-monitoring blood glucose test systems for over-the-counter use: Guidance for industry and food and drug administration staff” in 2020 and stated that the errors of a competing sensor must be less than 15% in reference to the test results from a standard glucometer, with 95% of all measurements satisfying such an error margin. These criteria must be satisfied before qualifying for other follow-up performance evaluations (and are hereafter referred to as the FDA accuracy benchmark). Prior to 2019, no noninvasive spectroscopic sensors had met this FDA accuracy benchmark until Chen et al. [17] suggested the addition of a pressure actuator to press on a patient’s finger at 20 N/cm^2^ against the attenuated total reflection (ATR) window of a Fourier-transform infrared (FTIR) spectrometer. Evidently, the applied pressure associated with this FTIR-based sensor effectively shortens the optical pathlength from the ATR window to the patient’s subcutaneous microcirculation network; thereby, the spectral signal-to-noise values are raised exponentially. The sensor’s configuration is depicted in Figure 1a.

Following the success of translating the fundamental physics in optical penetration and pathlength adjustment into improvements in noninvasive blood glucose measurements [17], we further extended this scientific research approach and yielded several technological advancements [18,19]. First, we revealed that common practices in correlating glucose concentration to the intensity of the highest glucose spectral peak at 1080 cm^−1^ erroneously neglect spectral interferences in this spectral region due to the inevitable presence of many other constituents in the interstitial fluid. By appropriately data-mining the spectral frame with the least spectral interference, we demonstrated that the accuracy of the sensor’s configuration, as shown in Figure 1a, can be enhanced without any hardware changes but with improvements in data-management software. More specifically, sampling the spectral intensities from 1000 to 1040 cm^−1^, with principal component analysis, the accuracy improvement is evident [20]. Second, we have also revealed that the common practice of collecting clinical blood glucose measurements from a patient’s finger pressed against the ATR window of an FTIR spectrometer has also not been thoroughly scrutinized. By tapping into the technological breakthrough in laser imaging subcutaneous blood and interstitial fluid flows, we have demonstrated that data collection from the hypothenar offers interstitial fluid signals stronger than data collection from the finger. Further, we advocate the logical upgrade of the common ATR to a multiple-pass-reflection version of such an ATR, i.e., with a multiple attenuation total reflection prism (MATR), to further enhance data collection [19]. In short, the first version of the FTIR-based sensor design shown in Figure 1a [17] has already been improved and presently transformed into a state-of-the-art sensor design [18]. The performance of this sensor is pictorially depicted with the standard Clarke error grid plot, as shown in Figure 1b, which proves clearly the confinement of all correlation data within the error grid zoning of error < 15%; as such, the sensor passes the FDA accuracy benchmark [18].

Although the state-of-the-art FTIR-based sensor design can satisfy the FDA accuracy benchmark, its exploitation in diabetes healthcare is practically barred because its bulkiness (like a large carry-on bag) and its measurement time (typically a few minutes) are both undesirable. Fortunately, the advance in mid-infrared quantum cascade lasers (QCLs) [21,22] can readily resolve these deficiencies. For example, a QCL-based sensor design for molecule detection in harsh environments in industry and a similar QCL-based sensor design for chemical detection in solutions in the healthcare sector, including detecting glucose in solution, were recently reported [23,24,25]. In spite of these promising advancements, no compact QCL-based sensor design for direct noninvasive glucose tests of patient-subjects has been disclosed.

Here, we disclose two high-performance designs of compact and accurate QCL-based glucose sensor systems for noninvasive clinical tests of blood glucose (hereafter referred as Sensor-System #1 and #2). In the following sections, we first elaborate on the common QCL-based sensor design elements which comprise a QCL emitting comb-like pulses from 8.9 to 10.7 µm to search for glucose, an MATR for interfacing with a patient-subject, and a computer-controlled infrared detector for sensing glucose. Noninvasive blood glucose measurements are conducted by having the patient’s hypothenar pressed against the MATR, with the detector giving glucose photoabsorptivity signals in the time domain. For Sensor-System #1, the conventional concept of spectroscopic analysis is retained in that the glucose photoabsorptivity signals are first translated to a glucose spectrum. For Sensor-System #2, the stereotype of spectroscopic analysis is abandoned and glucose concentrations are directly data-mined from the photoabsorptivity signals in the time domain.

## 2. Materials and Methods

### 2.1. Hardware Designs

**Figure 2:**
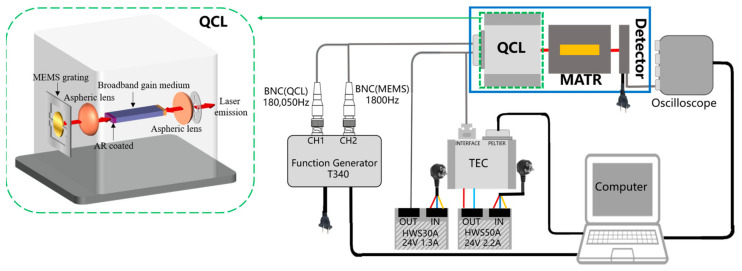
Schematic diagram of a QCL-based noninvasive blood glucose sensor.

Each of the two compact and accurate glucose sensors in the present work, as schematically illustrated in Figure 2, comprises a broadband (8.9 to 10.7 µm) pulsed QCL assembly (L14890-09, Hamamatsu Photonics K.K., Hamamatsu, Japan) with an average of 600 mW in full pulsed-power, a MATR, an infrared detector (C12494-011LH, Hamamatsu Photonics K.K., Hamamatsu, Japan), and a computer. The wavelength span of the QCL is equivalent to the wavenumber-span of 935 to 1128 cm^−1^, a span which adequately covers the critical spectral region of 1000 to 1040 cm^−1^ for the detection of glucose in blood with the least spectral interference [18]. The QCL assembly has a dimension of 82 mm in length, 81 mm in width, and 88 mm in height. In this assembly, a function generator (T340, Highland Technology, San Francisco, CA, USA) outputs one square-wave pulse train at 180,050 Hz to aid the QCL to emit pulses at this frequency, with another sine-wave train at 1800 Hz to turn a grating plate with a built-in microelectromechanical system to narrow the laser pulse width in time (and thus also in wavenumber) and raise the spectral resolving performance of each laser pulse. With these control components, the QCL assembly outputs 100 pulses in each grating-turning cycle time of 1/1800 s. Each pulse has an on-time of about 100 ns and an off-time of about 5.55 µs, with the first 50 pulses ramping up from 935 to 1128 cm^−1^ and the next 50 pulses ramping down from 1128 to 935 cm^−1^. The pulse-to-pulse separation is about 5.55 µs (and 3.86 cm^−1^). As such, an axisymmetric comb-like optical pulse train is generated, with pulse intensity as a function of time, and with time convertible to wavenumber.

**Figure 3:**
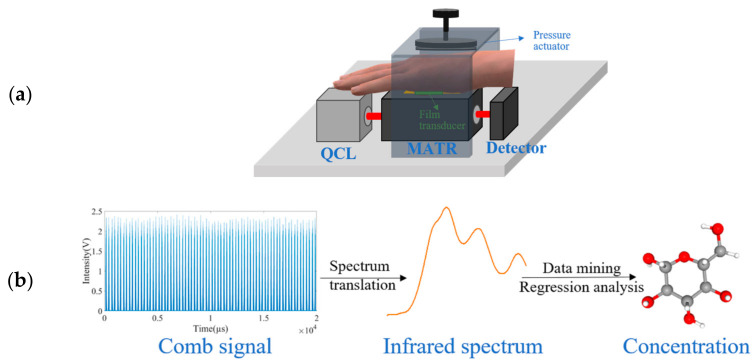
Schematics of the operation of QCL-Sensor-System #1: (**a**) Interface between the patient’s hypothenar and the sensor; (**b**) the route from raw comb signal, to comb-to-spectrum translation, and finally to statistical analysis of glucose concentration.

**Figure 4:**
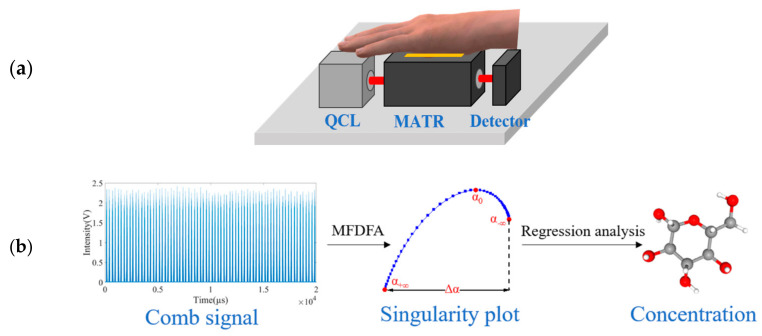
Schematics of the operation of QCL-Sensor-System #2: (**a**) Simplified interface hardware between the patient’s hypothenar and the sensor; (**b**) the simplified software route from generation of raw comb signal, to direct MFDFA data-mining of the four critical singularity strengths associated with MFDFA singularity plots, and finally to statistical analysis of glucose concentration.

The QCL pulses are coupled to a ZnSe MATR which is a trapezoid with a dimension of 72.4 mm in length and 5.7 mm in height, as shown in (Figure 3a and Figure 4a). The end faces are cut into facets of 45° in facet angle, to facilitate seven reflection passes on the upper surface. In a typical data acquisition process, a patient’s hypothenar is pressed against the MATR window of a QCL sensor with or without a pressure actuator. The contact pressures, measured with a film transducer, are typically 1–5 N/cm^2^ for a patient pressing the MATR window at ease, and are set to 20 ± 0.4 N/cm^2^ when a pressure actuator is added to the sensor. Optical signals are captured by a computer-controlled InAsSb infrared detector (C12494-011LH, Hamamatsu Photonics K.K., Hamamatsu, Japan), with dimensions of 65 mm in length, 20 mm in width, and 50 mm in height. The detector adequately covers a spectral response range from 2 to 11 μm. The analog signals from the detector are digitized and displaced with a compact and fast oscilloscope (Handyscope HS6 DIFF, TiePie Engineering, Sneek, The Netherlands) coupled to a common USB connector. Finally, the acquired data are transferred to a computer for data management. The FTIR-based sensor equipped with a MATR window and with a pressure actuator is constructed with a Nicolet IS50 FTIR spectrometer supplied by Thermo Fisher Scientific Inc., Shanghai, China. The details of this sensor and its performance have already been elaborated elsewhere [18].

### 2.2. Acquisition Software and Data-Mining Designs

In a typical testing operation, a comb-like train of emission pulses, with 100 pulses per 1/1800 s, is generated by the QCL assembly, and an associated comb-like train of data pulses is detected by the infrared detector, with the detected analog signals digitized by the USB oscilloscope which samples at a fast rate of 100 MHz, stores 20 million datapoints, and efficiently transfers this massive set of data in a batch to the computer. The acquisition is therefore much faster than a conventional spectrometer.

The present work further elaborates on two different modes of data management with a QCL sensor: comb-to-spectrum-translation mode and direct-comb-data-mining mode.

#### 2.2.1. Translation of Comb-like Pulses in Time to Infrared Spectrum in Wavenumber

In comb-to-spectrum-translation mode (Figure 3b), the conventional concepts in the spectral analysis of a chemical concentration in solution are adopted [26,27]. A method for comb-to-spectrum-translation is desirable because the emission-pulse train with 50 pulses, pulse width of 100 ns (in equivalent to approximately 0.07 cm^−1^), and pulse-to-pulse separation of about 5.55 µs (in equivalent to 3.86 cm^−1^) to cover a spectral range of 935 to 1128 cm^−1^ is very different from the conventional continuous emission source spectrum. Similarly, the raw photoabsorption data of the testing subject are merely a time series of narrow pulses each with a width of 0.07 cm^−1^ with a rather large wavenumber separation of 3.86 cm^−1^ in which no spectral signal is present; thus, comb-like photoabsorption data in a time series are also very different from the conventional photoabsorption spectrum readily available for the estimation of a chemical concentration from spectral peak intensities. A solution to resolve this problem is to increase the density of narrow pulses high enough that the tips of the pulses seemingly outline a continuous spectral profile. Upon this, the conversion of the time coordinate to the wavenumber coordinate gives a conventional absorption spectrum.

**Figure 5:**
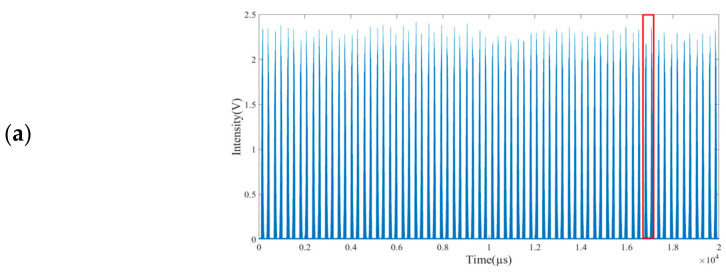

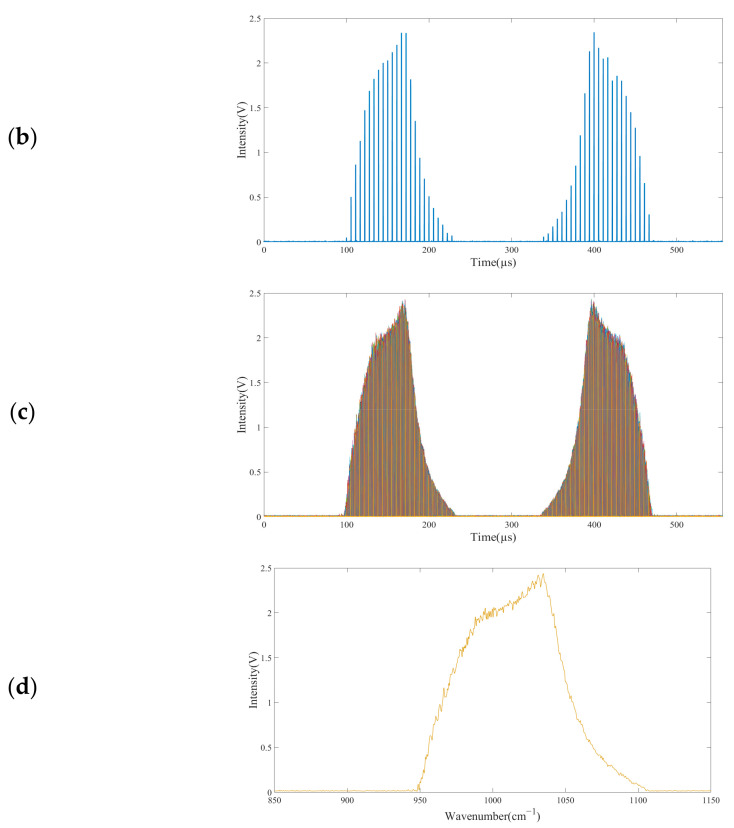
Conversion of raw data to continuous line spectrum (envelope). (**a**) Raw time signals of 36 full cycles. (**b**) An example showing 1 (in the red box) of these 36 full cycles. (**c**) Overlapping result for 36 full cycles. (**d**) A continuous emission spectrum from merging these two axisymmetric spectra of (**c**).

More specifically, in one cycle time of 1/1800 s, the angle of the optical grating in the QCL assembly is turned clockwise and then counterclockwise for a complete period such that 100 broadband QCL pulses are sharpened to a train of 100 narrow pulses each with a full pulse width of 100 ns (equivalent to 0.07 cm^−1^), and with the first 50 ramping up from 935 to 1128 cm^−1^ and the next 50 ramping down from 1128 cm^−1^ back to 935 cm^−1^ (hereafter referred as a full cycle). Readily shown in Figure 2, the cycle time for the QCL to emit 100 pulses is purposedly set at 1/1800 s which is asynchronously off by 0.154 µs (1/36 of the pulse-to-pulse separation) from the full cycle time of 555.555 µs in which 100 pulses are emitted by the QCL. Due to this exact asynchronization setting, the emission pulses of one full cycle are all off by 0.154 µs (equivalent to 0.107 cm^−1^) relative to those of the preceding full cycle. As such, although for each single half-cycle, the emission-pulse train has merely 50 narrow pulses each with a pulse width of 0.07 cm^−1^ and a large pulse-to-pulse separation of 3.86 cm^−1^, and the combined emission-pulse train of 36 full cycles comprises 3600 pulses in 386 cm^−1^ (one train from 935 to 1128 cm^−1^ and another train from 1128 cm^−1^ back to 935 cm^−1^). The condensed train thus comprises pulses with a pulse width of 0.07 cm^−1^ and with a very small pulse-to-pulse separation of 0.107 cm^−1^. The pulse train in time for 36 full cycles is shown in Figure 5a, with the pulse train in time for 1 of these 36 full cycles shown in Figure 5b. Merging the pulse trains of 36 full cycles into 1 full cycle yields two axisymmetric emission spectra as shown in Figure 5c. A virtually continuous emission spectrum is built by merging these two axisymmetric spectra as shown in Figure 5d.

In all practical measurements, a long comb-like pulse train in time, with a total time of 10 × 36/1800 s, is collected and used to produce the average pulse train behavior in a full-time cycle of 36/1800 s (hereafter referred as an “average comb”). Similarly, an average emission spectrum is produced by averaging 10 emission spectra. When a sample is tested with the QCL emissions, an average raw photoabsorption spectrum is produced with the same methodology of comb-to-spectrum translation, obviously by passing the QCL emissions through the test sample to the infrared detector.

**Figure 6:**
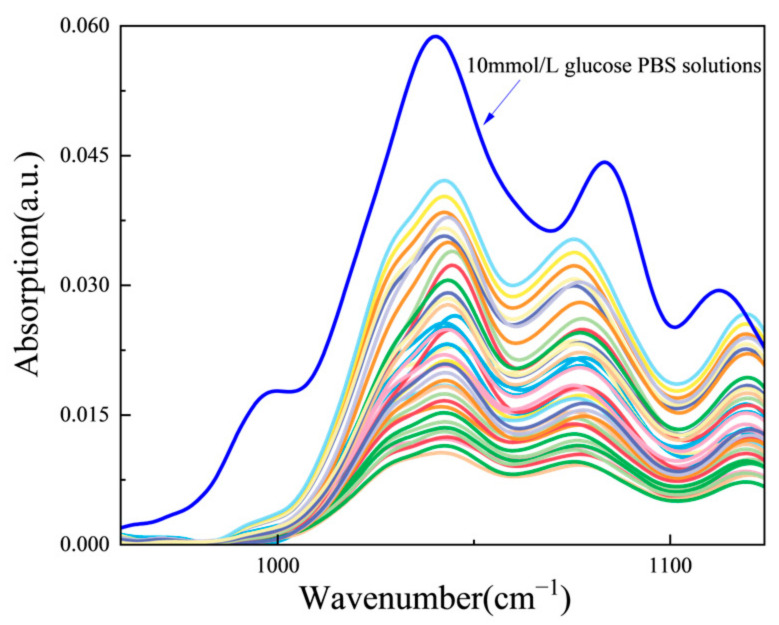
The comb-to-spectrum-translation results of 41 photoabsorption spectra (painted in color) from Patient-Subject #1, with a photoabsorption spectrum (painted in blue) collected by an FTIR spectrometer as a reference.

Since the spectral intensity of the QCL emission spectrum is not wavenumber independent, each raw photoabsorption spectrum must be normalized with the average emission spectral data, for the production of a photoabsorption spectrum which is expected to compare well with the photoabsorption spectrum produced by an FTIR spectrometer. A QCL photoabsorption spectrum of glucose in a phosphate-buffered solution (PBS) is compared, in Figure 6, with that collected with a Nicolet IS50 FTIR spectrometer, to experimentally validate this claim.

With this validity, the translation of combs from patient-subjects having variable amounts of blood glucose to glucose photoabsorption spectra is produced, as shown in Figure 6. The glucose photoabsorption spectra from QCL are virtually the same as their FTIR counterparts. Following this, the spectral data are first analyzed with the principal component analysis (PCA) method to minimize spectral interferences and other error sources; then, the PCA results are further analyzed with the support vector machine (SVM) regression method to yield a model for finding glucose concentrations from glucose photoabsorption spectra. The details of the PCA and SVM data management have already been reported elsewhere [18].

#### 2.2.2. Direct Combs Data-Mining via Multifractal Detrended Fluctuation Analysis

The preceding section elaborates on the translation of a train of pulses in time collected from a patient-subject to a conventional photoabsorption spectrum of blood glucose. This translation is practical because such a train of pulses in time is critically influenced by the concentration of glucose. In other words, the concentration of glucose is an intrinsic property of the pulses in a time series. Here, we hypothesize that multifractal detrended fluctuation analysis (MFDFA) [28] is applicable to extract the glucose concentration from comb-like pulses in time as depicted in Figure 4b, because MFDFA has been successfully used to extract intrinsic properties from data in a time series. Exemplars of this success include extracting multiple risk factors from time changes in pricing of goods and associated business attributes, and extracting entangled tool-failure factors from time measurements of strains and vibrations [29,30].

To articulate our hypothesis, we cite the outstanding work on tool condition monitoring in milling processes using MFDFA and SVM by Guo et al. [29], as a good case study to explain the methodology of MFDFA and SVM. Briefly, cutting forces and vibrations, in time series, are recorded and subjected to MFDFA. A quantity known as the fractal dimension, *f*(*α*), as a function of another quantity known as the singularity exponent, *α*, is computed in such MFDFA applications. From the plot of *f*(*α*) against *α*, known as a singularity spectrum, three primary critical singularity strengths, *α*_+∞_ (the leftmost point of the singularity spectrum), *α*_−∞_ (the rightmost point of the singularity spectrum), and *α*_0_ (the peak of *f*(α)) are extracted. Additional secondary critical singularity strengths are formulated with these three primary critical singularity strengths. All of these critical singularity strengths are then analyzed by the SVM regression method to pin down the intrinsic properties of the data-trains in time. In the case of the work by Guo et al., seven critical singularity strengths are extracted and the SVM results facilitate accurate classification of the tested tool conditions with an accuracy of 95.6% [29]. In the present work on direct comb data-mining of glucose concentrations, combs are subjected to MFDFA and four critical singularity strengths, *α*_+∞_, *α*_−∞_, *α*_0_, and Δ*α* (defined as *α*_−∞_–*α*_+∞_) are extracted from each concentration test of the 41 concentration tests collected from each patient-subject of four patient-subjects. The critical singularity strengths are analyzed by the SVM method to deduce a model for computing a concentration from a set of critical singularity strengths. The detailed theory and mathematical formulations in MFDFA are included in the Supplementary Materials.

#### 2.2.3. Regression Analysis Method

SVM is a powerful classification tool developed by Vapnik et al. based on the statistical learning theory [31]. In the present work, for Sensor-System #1, the well-established PCA is used to reduce the dimensionality of the data with the resultant datasets further analyzed via SVM. Meanwhile, for Sensor-System #2, the critical singularity strengths extracted from the singularity spectra are analyzed by SVM. In this work, the MATLAB (R2023b) Regression Learner application package and a graphical user interface of MATLAB’s Statistics and machine-learning toolbox are employed to train data and to predict data for the SVM model. In addition, to verify the generalization ability of the SVM model, a 5-fold cross validation is performed on the training data.

#### 2.2.4. Clarke Error Grid Analysis

The new testing techniques using the noninvasive sensor systems developed in this work are assessed by the correlation of test results with those from a standard glucometer. The Clarke error grid method [32] is a prevalent graphic analysis of correlation and recognized as a standard correlation tool in the ISO15197 standard [33] for clinal blood glucose measurements. In the Clarke error grid method, paired measurements by the two different methods are included in a Clarke error grid plot on which zones are catalogized by the errors of correlation. As such, a visual view of the locations of plotted data in reference to these error zones in a Clarke error grid plot aids the correlation assessment of the two methods. In the present work, paired data (blood analysis with a glucometer and spectral analysis with a noninvasive QCL sensor system) are placed in a Clarke error grid plot, and data falling in Zone A in the grid (correlation errors being less than 20%) are adopted as a convenient preliminary correlation benchmark. In addition, a gray grid with correlation errors being less than 15% is added in each Clarke error grid plot for matching the FDA accuracy benchmark which requires 95% of correlation data to be inside the 15% error grid.

### 2.3. Experimental Procedure

In this work, the sensor accuracy is assessed and validated with glucose measurements recorded from a group of patients having variable blood glucose concentrations, in compliance with the standard “Oral Glucose Tolerance Test” recognized by the American Diabetes Association for cross-checking the diabetic condition of a patient. The sensor accuracy is benchmarked to those from a standard glucometer. The following experimental procedures are designed in accordance with other recent assessments and validations of noninvasive blood glucose methods [34,35].

1. Specifically, 4 healthy and nondiabetic patient-subjects, 2 males and 2 females, conducting the tests are requested to fast for 10–12 h before 8 am every day. At the end of the fasting period, true blood glucose values are first detected and recorded with a standard glucometer. Then, noninvasive blood glucose measurements are conducted using Sensor-System #1 and Sensor-System #2 by taking 3 QCL scans from each of the four patient-subjects. The blood glucose concentrations thus measured are expected to near the normal “heathy” level of 3.9 mM.
2. Afterwards, each of the four patient-subjects is requested to orally intake 75 g glucose and is then asked to accept a sequence of glucose tests to track the blood glucose variations. The sequence comprises a set of 12 tests in a total log time of 120 min, with a time duration of 10 min between two consecutive tests. The measured blood glucose concentrations exceeding 7.9 mM are discarded and nominally 41 concentrations ranging from 3.9 to 7.9 mM, with an increment of 0.1 mmol/L, are measured and recorded.
3. For every set, each patient-subject is requested to take the quick sensor measurements 9 min after the finger-pricking step of the standard test with a glucometer. This time lag of 9 min is prescribed because the finger-pricking test measures glucose in the bloodstream, while the noninvasive sensors detect glucose in the interstitial fluid, and the diffusion of glucose from the bloodstream to the interstitial fluid is known to have an average time lag of about 8–10 min [34,35]. Here, it should be noted that tests employing Sensor-System #1 and Sensor-System #2 are completed at a rate of about one second per test; hence, the glucose variations in time are negligible for such a short time difference.
4. Each noninvasive blood glucose test is conducted by requesting each patient-subject to press the subject’s hypothenar at ease against the MATR window of the sensor. A built-in pressure transducer records a pressure range of 1–5 N/cm^2^. To further enhance its accuracy, Sensor-System #1 is equipped with a pressure actuator to mechanically press the patient’s hypothenar against the MATR window at a firm pressure of 20 ± 0.4 N/cm^2^. Again, for each test, 3 QCL scans are taken.

In the present work, five repetitive “Oral Glucose Tolerance Test” processes are administrated for 2 males and 2 females, all of them nondiabetic. All participants gave their verbal consent.

## 3. Results and Discussion

### Assessment and Validation of Sensor Accuracy

With Sensor-System #1, each set of raw comb signals is translated to a glucose spectrum. For example, 41 spectra for one of the four patient-subjects are summarized in Figure 6. The spectral data within 1000 to 1040 cm^−1^ of each spectrum are treated with the PCA method to reduce their dimensionality and with the SVM regression method to statistically yield a glucose concentration.

**Figure 7:**
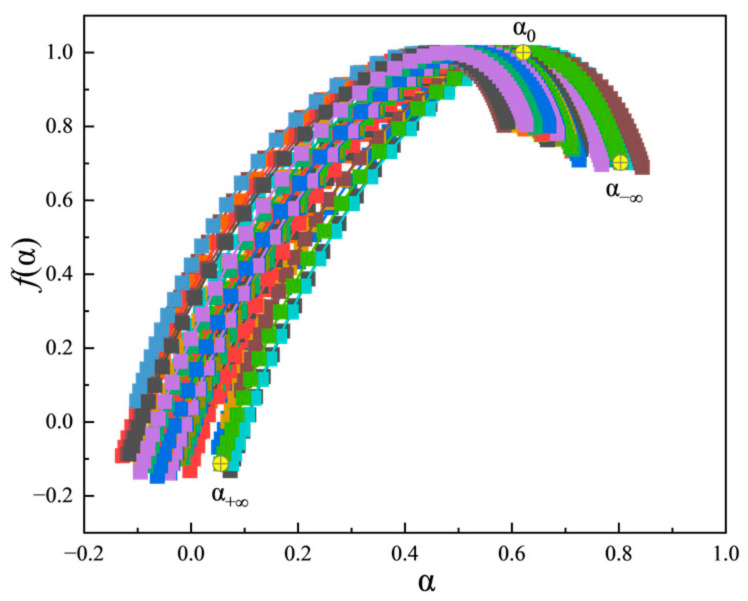
The singularity spectra generated by MFDFA of the same 41 comb trains the comb-to-spectrum translation of which give the 41 photoabsorption spectra shown in Figure 6.

With Sensor-System #2, each comb-like train of pulses in time collected from every glucose test is directly treated with the MFDFA method, and the resultant critical singularity strengths are treated with the SVM regression method to statistically yield a glucose concentration. The singularity spectra from the same 41 glucose tests giving conventional spectra in Figure 6 are summarized in Figure 7. It should be noted that the singularity spectra in Figure 7 are fundamentally different from the conventional photoabsorption spectra in Figure 6. A conventional photoabsorption spectrum shows spectral intensity as a function of wavenumber, and a statistical analysis of spectral intensity gives a test concentration. As for the singularity spectrum, a function of the fractal dimension is plotted again the singularity strength, and a statistical analysis of the fractal dimension does not give a test concentration. Instead, a statistical analysis of the primary critical singularity strengths extracted from the singularity spectrum and additional secondary critical singularity strengths derived from the primary critical singularity strengths gives a test concentration.

It should also be clarified that in this work, for each sensor-testing operation conducted with one patient-subject, three QCL scans are collected for each of the 41 concentration variations; hence, 123 datasets are collected. With these, 90 datasets are used to form the training set and 33 datasets are used to form the testing set. The SVM regression analysis is conducted using a quadratic polynomial kernel function in conjunction with a 5-fold cross-validation for the training set. Finally, the performance of the regression model is evaluated with an independent testing set.

**Figure 8:**
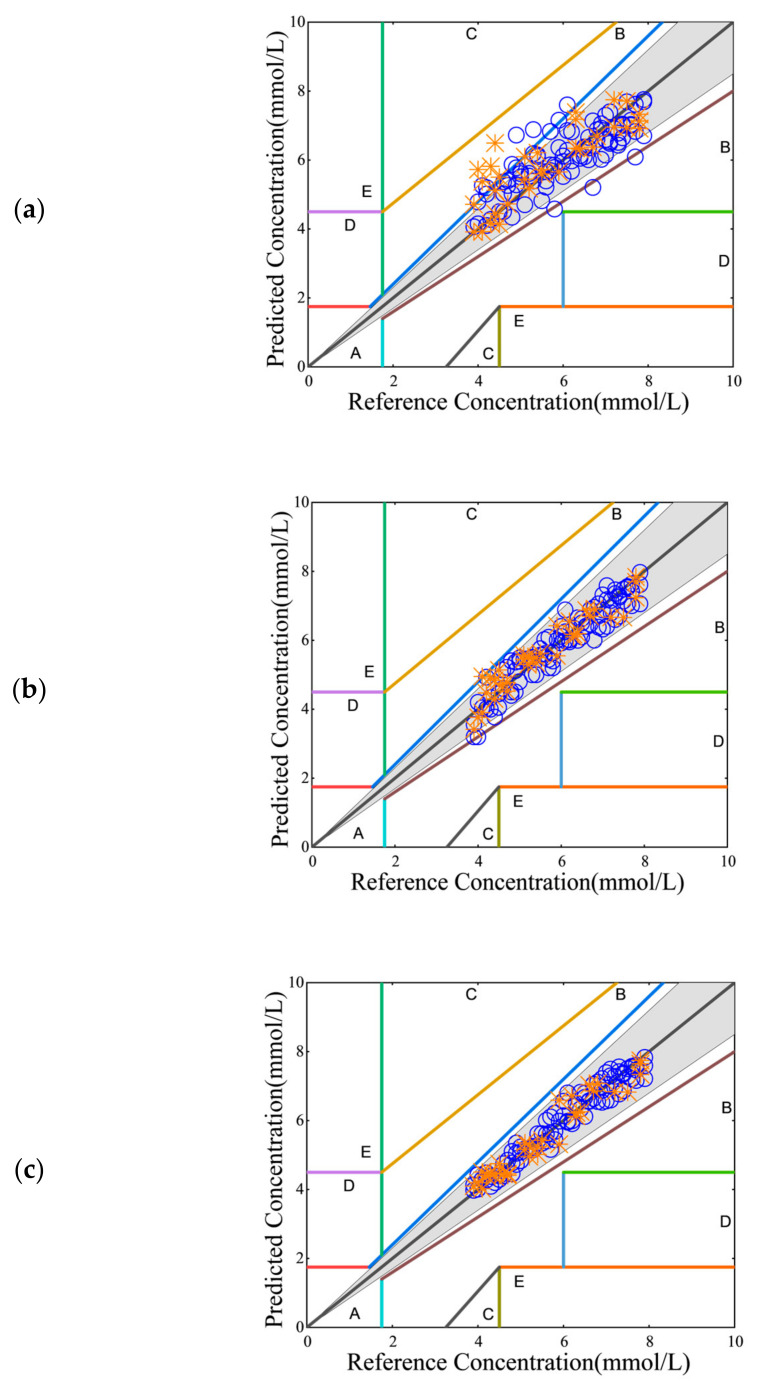
Clarke error grid plots corresponding to (**a**) Sensor-System #1 without a pressure actuator; (**b**) Sensor-System #1 with a pressure actuator; (**c**) Sensor-System #2 without a pressure actuator. (The blue circles are the data of the training set, and the orange crosses are the data of the testing set.)

The noninvasive testing results in this work are found to correlate well with those from the glucometer. For example, the Clarke error grid plots showing the performance of Sensor-System #1 and Sensor-System #2 for Patient-Subject #1 are summarized in Figure 8. Briefly, for Sensor-System #1 without a pressure actuator, the correlation data are scattered, as shown in Figure 8a. A significant portion of them lies outside the gray zone for error < 15%. More precisely, 18.9% of measurements in the training set and 30.3% of measurements in the testing set are outside the gray zone. Clearly, Sensor-System #1 without a pressure actuator fails to meet the FDA accuracy benchmark (<5% outside the gray zone) for replacing a standard glucometer. In comparison, 97% of measurements conducted with Sensor-System #1 equipped with a pressure actuator for assuring a firm contact pressure of 20 N/cm^2^ fall inside the gray zone, as shown in Figure 8b; thus, this sensor system surpasses the FDA accuracy benchmark. As anticipated in the present work, measurements conducted with Sensor-System #2 having no pressure actuator are evidently more accurate than Sensor-System #1 having a pressure actuator. The corresponding Clark error grid plot, as shown in Figure 8c, proves that all measurements with Sensor-System #2 having no pressure actuator fall in the gray zone, and thus meet the FDA accuracy benchmark.

The plots for the other three patient-subjects are included in Figures S5–S7 of the Supplementary Materials. The overall sensor performances for all sensors and all patient-subjects are summarized in Table 1. Evidently, Sensor-System #1 equipped with a pressure actuator and Sensor-System #2 meet the FDA accuracy benchmark for all patient-subjects. Statistically, Sensor-System #2 outperforms Sensor-System #1 equipped with a pressure actuator. These two sensor-systems and the FTIR-based sensor are further compared in size and weight, and the results are summarized in Table S1 in the Supplementary Materials. Evidently, Sensor-System #2 is an outstanding design for practical noninvasive blood glucose measurements, with value propositions good enough for substituting standard glucometers. The nominal test time for Sensor-System #2 is 1–10 s, and that for an FTIR-based sensor is about 10 min. We expect that future advancements in QCL fabrication, production, and marketing will further shrink the size, weight, and cost of this prototype sensor system, and that the future integration of QCL, MATR, and IR detectors into a monolithic system as convenient as a cellphone will eventually be developed and exploited for improving diabetes healthcare management.

## 4. Conclusions

A prototype of a practical compact QCL-based noninvasive glucose sensor system has been developed. It comprises a QCL unit, a MATR, an infrared detector, and a computer-based data management unit, with a size and weight more suitable for portable applications than an FTIR-based design. The comb-like pulses in time emitted by a QCL equipped with a MEMS-controlled grating, after passing the MATR interfaced with a patient-subject, are directly subjected to MFDFA for the generation of a set of singularity spectra. From the singularity spectra, critical singularity strengths are extracted and transformed to concentration data via advanced statistical analysis including machine learning. This noninvasive glucose sensor is fast and convenient, with its accuracy passing the FDA accuracy benchmark. With future research on sensor miniaturization, effective production, marketing, sales, and customer satisfaction, we opine that this sensor design can likely be developed to a compact noninvasive glucometer for replacing the outdated and inconvenient finger-pricking sensing products in the market.

## Figures and Tables

**Table 1:** Sensor performance: % of measurements with errors not more than the listed value.

Subjects	Sensors	*w*/*wo* Pressure Actuator	% of Measurements	
			Error ± 15%	Error ± 20%
Subject 1	Sensor-System #1	without pressure actuator	75.41	85.36
		with pressure actuator	96.97	98.49
	Sensor-System #2	without pressure actuator	100.00	100.00
Subject 2	Sensor-System #1	without pressure actuator	74.45	85.36
		with pressure actuator	98.34	98.34
	Sensor-System #2	without pressure actuator	99.45	100.00
Subject 3	Sensor-System #1	without pressure actuator	78.99	83.44
		with pressure actuator	98.34	98.34
	Sensor-System #2	without pressure actuator	100.00	100.00
Subject 4	Sensor-System #1	without pressure actuator	79.55	88.94
		with pressure actuator	97.62	100.00
	Sensor-System #2	without pressure actuator	99.45	100.00
Average	Sensor-System #1	without pressure actuator	77.10	85.78
		with pressure actuator	97.82	98.79
	Sensor-System #2	without pressure actuator	99.73	100.00

## Data Availability

The dataset generated and analyzed during this study is available from the corresponding author upon reasonable request, but restrictions apply to the commercially confidential details.

## Supplementary Materials

The following supporting information can be downloaded at https://www.mdpi.com/article/10.3390/s25020587/s1. Table S1: Size and weight of an FTIR vs those of a QCL sensor; Table S2: Singularity strengths of Patient-Subject #1; Figure S1: The photoabsorption spectra generated by the combs-to-spectrum-translation method for (a) Patient-Subject #2; (b) a Patient-Subject #3; (c) Patient-Subject #4, all tested with Sensor-System #1 with a pressure actuator set to a nominal contact-pressure of 20N/cm2; for each patient-subject, one spectrum is generated for one glucose concentration and a total of 41 spectra are shown; Figure S2: The photoabsorption spectra generated by the combs-to-spectrum-translation method for (a) Patient-Subject #1; (b) a Patient-Subject #2; (c) Patient-Subject #3; and (d) Patient-Subject #4, all tested with Sensor-System #1 with no pressure actuator; for each patient-subject, one spectrum is generated for one glucose concentration and a total of 41 spectra are shown; Figure S3: The singularity spectra directly deduced via MFDFA from the raw combs, with 41 singularity spectra for each patient-subject, (a) Patient-Subject #2; (b) a Patient-Subject #3; (c) Patient-Subject #4; Figure S4: The correlation plots of singularity strengths vs glucose concentrations: (a) α_+∞_, (b) α_0_, (c) α_−∞_, (d) Δα for Patient-Subject #1; Figure S5: Clarke error grid plots corresponding to (a) Sensor-System #1 without pressure actuator; (b) Sensor-System #1 with pressure actuator; (c) Sensor-System #2 without pressure actuator for Patient-Subject #2; Figure S6: Clarke error grid plots corresponding to (a) Sensor-System #1 without pressure actuator; (b) Sensor-System #1 with pressure actuator; (c) Sensor-System #2 without pressure actuator for Patient-Subject #3; Figure S7: Clarke error grid plots corresponding to (a) Sensor-System #1 without pressure actuator; (b) Sensor-System #1 with pressure actuator; (c) Sensor-System #2 without pressure actuator for Patient-Subject #4.

## References

[1] Elsayed N.A., Aleppo G., Aroda V.R., Bannuru R.R., Brown F.M., Bruemmer D., Collins B.S., Hilliard M.E., Isaacs D., Johnson E.L. (2023). Classification and Diagnosis of Diabetes: Standards of Care in Diabetes-2023. Diabetes Care.

[2] (2023). Noncommunicable Diseases. https://www.who.int/news-room/fact-sheets/detail/noncommunicable-diseases.

[3] So H.C., Wong T.K., Chung J. (2012). Recent advances in noninvasive glucose monitoring. Med. Devices.

[4] Oliver N.S., Toumazou C., Cass A.E.G., Johnston D.G. (2009). Glucose sensors: A review of current and emerging technology. Diabet. Med..

[5] Vashist S.K. (2012). Non-invasive glucose monitoring technology in diabetes management: A review. Anal. Chim. Acta.

[6] Thennadil S.N., Rennert J.L., Wenzel B.J., Hazen K.H., Ruchti T.L., Block M.B. (2004). Comparison of glucose concentration in interstitial fluid, and capillary and venous blood during rapid changes in blood glucose levels. Diabetes Technol. Ther..

[7] Steil G.M., Rebrin K., Hariri F., Jinagonda S., Tadros S., Darwin C., Saad M.F. (2005). Interstitial fluid glucose dynamics during insulin-induced hypoglycaemia. Diabetologia.

[8] Kim H., Noh I., Yoon G. (2009). Glucose Prediction in the Interstitial Fluid Based on Infrared Absorption Spectroscopy Using Multi-component Analysis. J. Environ. Biol..

[9] Kino S., Omori S., Katagiri T., Matsuura Y. (2016). Hollow optical-fiber based infrared spectroscopy for measurement of blood glucose level by using multi-reflection prism. Biomed. Opt. Express.

[10] Matsuura Y., Koyama T., Razeghi M., Lewis J.S., Khodaparast G.A., Tournié E. (2019). Non-invasive blood glucose measurement using quantum cascade lasers. Quantum Sensing and Nano Electronics and Photonics XVI.

[11] Kong C.R., Barman I., Dingari N.C., Kang J.W., Galindo L., Dasari R.R., Feld M.S. (2011). A novel non-imaging optics based Raman spectroscopy device for transdermal blood analyte measurement. Aip Adv..

[12] Shih W.C., Bechtel K.L., Rebec M.V. (2015). Noninvasive glucose sensing by transcutaneous Raman spectroscopy. J. Biomed. Opt..

[13] Aloraynan A., Rassel S., Xu C., Ban D. (2022). A Single Wavelength Mid-Infrared Photoacoustic Spectroscopy for Noninvasive Glucose Detection Using Machine Learning. Biosensors.

[14] Pleitez M.A., Lieblein T., Bauer A., Hertzberg O., Von Lilienfeld-Toal H., Mantele W. (2013). In Vivo Noninvasive Monitoring of Glucose Concentration in Human Epidermis by Mid-Infrared Pulsed Photoacoustic Spectroscopy. Anal. Chem..

[15] Yu Y., Huang J.-P., Zhu J., Liang S.-L. (2020). An Accurate Noninvasive Blood Glucose Measurement System Using Portable Near-Infrared Spectrometer and Transfer Learning Framework. IEEE Sens. J..

[16] Tanaka Y., Tajima T., Seyama M., Waki K. (2020). Differential Continuous Wave Photoacoustic Spectroscopy for Non-Invasive Glucose Monitoring. IEEE Sens. J..

[17] Chen J.Y., Zhou Q., Xu G., Wang R.T., Tai E.G., Xie L., Zhang Q., Guan Y., Huang X. (2019). Non-invasive blood glucose measurement of 95% certainty by pressure regulated Mid-IR. Talanta.

[18] Song L., Han Z., Lau W.-M. (2024). Optimization of mid-infrared noninvasive blood-glucose prediction model by support vector regression coupled with different spectral features. Spectrochim. Acta A Mol. Biomol. Spectrosc..

[19] Song L., Han Z., Shum P.-W., Lau W.-M. (2024). Enhancing the accuracy of blood-glucose tests by upgrading FTIR with multiple-reflections, quantum cascade laser, two-dimensional correlation spectroscopy and machine learning. Spectrochim. Acta A Mol. Biomol. Spectrosc..

[20] He Z., Liu Y. (2021). Fourier Transform Infrared Spectroscopic Analysis in Applied Cotton Fiber and Cottonseed Research: A Review. J. Cotton Sci..

[21] Yao Y., Hoffman A.J., Gmachl C.F. (2012). Mid-infrared quantum cascade lasers. Nat. Photonics.

[22] Ma Y.H., Ding K.K., Wei L., Li X., Shi J.C., Li Z.J., Qu Y., Li L., Qiao Z.L., Liu G.J. (2022). Research on Mid-Infrared External Cavity Quantum Cascade Lasers and Applications. Crystals.

[23] Teuber A., Mizaikoff B. (2024). Robust Attenuated Total Reflection Infrared Spectroscopic Sensors Based on Quantum Cascade Lasers for Harsh Environments. IEEE Sens. J..

[24] Brandstetter M., Volgger L., Genner A., Jungbauer C., Lendl B. (2013). Direct determination of glucose, lactate and triglycerides in blood serum by a tunable quantum cascade laser-based mid-IR sensor. Appl. Phys. B.

[25] Jernelv I.L., Strøm K., Hjelme D.R., Aksnes A. (2019). Infrared Spectroscopy with a Fiber-Coupled Quantum Cascade Laser for Attenuated Total Reflection Measurements Towards Biomedical Applications. Sensors.

[26] Koyama T., Shibata N., Kino S., Sugiyama A., Akikusa N., Matsuura Y. (2020). A Compact Mid-Infrared Spectroscopy System for Healthcare Applications Based on a Wavelength-Swept, Pulsed Quantum Cascade Laser. Sensors.

[27] Chen J., Furukawa H. (2022). High-speed mid-infrared spectrometer based on wavelength-swept quantum cascade laser using asynchronous-signal method. Opt. Laser Technol..

[28] Kantelhardt J.W., Zschiegner S.A., Koscielny-Bunde E., Havlin S., Bunde A., Stanley H.E. (2002). Multifractal detrended fluctuation analysis of nonstationary time series. Phys. A Stat. Mech. Appl..

[29] Guo J., Li A., Zhang R. (2020). Tool condition monitoring in milling process using multifractal detrended fluctuation analysis and support vector machine. Int. J. Adv. Manuf. Technol..

[30] Guo Y., Zhang S., Liu Y. (2022). Research on Risk Features and Prediction of China’s Crude Oil Futures Market Based on Machine Learning. Front. Energy Res..

[31] Vapnik V., Vapnik V., Vapnik V.N. (1995). The Natural of Statistical Learning Theory.

[32] Clarke W.L., Cox D., Gonder-Frederick L.A., Carter W., Pohl S.L. (1987). Evaluating clinical accuracy of systems for self-monitoring of blood glucose. Diabetes Care.

[33] (2013). In Vitro Diagnostic Test Systems—Requirements for Blood-Glucose Monitoring Systems for Self-Testing in Managing Diabetes Mellitus.

[34] Chuah Z.-M., Paramesran R., Thambiratnam K., Poh S.-C. (2010). A two-level partial least squares system for non-invasive blood glucose concentration prediction. Chemom. Intell. Lab. Syst..

[35] Yang W., Liao N., Cheng H., Li Y., Bai X., Deng C. (2018). Determination of NIR informative wavebands for transmission non-invasive blood glucose measurement using a Fourier transform spectrometer. AIP Adv..

