# Supplementary material for: Compact Quantum Cascade Laser-Based Noninvasive Glucose Sensor Upgraded with Direct Comb Data-Mining
Source: Sensors (Basel). 2025 Jan 20;25(2):587. doi: 10.3390/s25020587 (PMC11768889; doi:10.3390/s25020587)
Supplement: Supplementary file 1 [file sensors-25-00587-s001.zip › sensors-3346167-supplementary.pdf]

Supplementary Materials

Compact Quantum Cascade Laser-Based Noninvasive Glucose Sensor Upgraded with Direct Comb Data-Mining

Liying Song [1,2], Zhiqiang Han [3,4,5,*], Hengyong Nie [3,6,7] and Woon-Ming Lau [2,3,4,5,*]

[1] School of Mathematics and Physics, University of Science and Technology Beijing, Beijing 100083, China; songliying.sly@foxmail.com
[2] Shunde Innovation School, University of Science and Technology Beijing, Foshan 528399, China
[3] School of Chemistry and Chemical Engineering, Linyi University, Linyi 276000, China; hnie@uwo.ca
[4] The Sun Age New Energy Ltd., Zhuhai 519100, China
[5] The Yaoling Age New Energy Technology Development Ltd., Linyi 276000, China
[6] Surface Science Western, Western University, London N6G 0J3, Canada
[7] Department of Physics and Astronomy, Western University, London N6A 3K7, Canada
* Correspondence: hanzhiqiang@lyu.edu.cn (Z.H.); leolau@lyu.edu.cn (W.M.L.)

Size and weight of an FTIR vs those of a QCL sensor

The size and weight of an FTIR are much larger than those of a QCL sensor. The corresponding data are listed in Table S1.

Table S1. Size and weight of an FTIR vs those of a QCL sensor

**Equipment/device**		**Physical parameters**	
		L(mm)×W(mm)×H(mm)	Weight(kg)
FTIR spectrometer	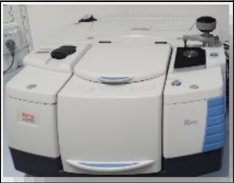	850×750×400	70.00
QCL	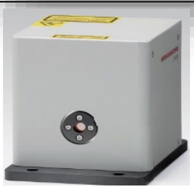	112×82×88	1.20
MATR	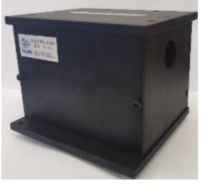	115×100×90	0.90
Detector	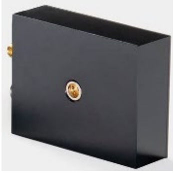	65×20×50	0.09
Function generator	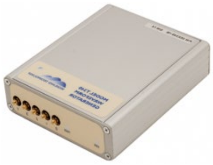	48×40×12	0.20
TEC	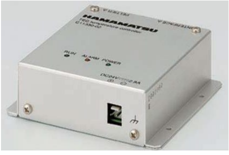	110×100×33	0.30
Oscilloscope	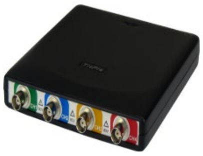	150×120×25	0.5

Multifractal detrended fluctuation analysis method

The method of multifractal detrended fluctuation analysis (MFDFA; [1]) has been successfully applied to extract intrinsic properties of data in time series for applications such as finding multiple risk factors from historical changes in pricing of goods and associated business attributes, and finding entangled tool-failure factors in time measurements of strains and vibrations. As such, MFDFA is adopted in the present work to extract information on glucose concentrations from the raw comb-like pulse trains in time taken from patient-subjects dosed with various amounts of glucose. In this section, the basic procedure for MFDFA of nonstationary time series x_k (k =1, 2, ..., N) is as follows:

1. Determine the profile $Y(i)$ as a function of the time variable x_k

$$Y(i) = \sum_{k=1}^{i} (x_k - \langle x \rangle), i = 1, \dots, N \tag{S1}$$

$$\langle x \rangle = \frac{1}{N} \sum_{k=1}^{N} x_k \tag{S2}$$

2. Divide the profile $Y(i)$ into a number (N_s) of nonoverlapping segments of equal length s. Since the full-length of $Y(i)$ is often not exactly N_s*s, a short part at the end of the profile may remain outside the coverage by these N_s segments. In order to fully utilize all data (full-length of $Y(i)$, the same procedure is repeated starting from the opposite end. Thereby, $2N_s$ segments are obtained.

3. Calculate the local trend for each of the $2N_s$ segments by a least-square fit of the series. Then determine the variance

$$F^2(v, s) = \sum_{i=1}^{s} \{Y[(v-1)s + i] - y_v(i)\}^2, v = 1, \dots, N_s, \tag{S3}$$

$$F^2(v, s) = \sum_{i=1}^{s} \{Y[N - (v - N_s)s + i] - y_v(i)\}^2, v = N_s + 1, \dots, 2N_s \tag{S4}$$

Here, $y(i)$ is the fitting trend in v^{th} segment.

4. Average over all segments to obtain the qth-order fluctuation function $F_q(s)$:

$$F_q(s) = \left\{ \frac{1}{2N_s} \sum_{v=1}^{2N_s} [F^2(v, s)]^{q/2} \right\}^{1/q} \tag{S5}$$

5. If the time series x_k exhibits self-similarity features, there is a power-law relationship between the average of the qth-order fluctuation function $F_q(s)$ and the time scale s

$$F_q(s) \sim s^{h(q)} \tag{S6}$$

where $h(q)$ is the generalized Hurst exponent or self-similarity exponent. The generalized Hurst exponent $h(q)$ is obtained by finding the slope of the $F_q(s)$ curve in the log-log plots. For multifractal data, the dependence on q can be understood by studying the multifractal scaling exponent $\tau(q)$, given by

$$\tau_q = qh(q) - 1, \tag{S7}$$

which depends on the generalized Hurst exponent $h(q)$. Similarly, one can construct the singularity-spectrum $f(\alpha)$ via Legendre transform. If $\tau(q)$ is sufficiently smooth, the singularity-strength α is given by

$$\alpha = \tau'(q) = h(q) + qh'(q), \tag{S8}$$

from which the singularity-spectrum $f(\alpha)$ can be constructed as

$$f(\alpha) = q\alpha - \tau_q. \tag{S9}$$

The singularity-spectrum $f(\alpha)$ describes the dimension of the subset of the time series which is characterized by the singularity strength α.

Measurement results from patient-subjects and data-analysis

In the design of the research in this work, four patient-subjects participate in conducting measurements with a glucometer, a modified FTIR-based sensor, a QCL-based sensor with a pressure actuator, and a QCL-based sensor without a pressure actuator. Each patient-subject is requested to take 41 different glucose dosages, and when the glucose concentration is confirmed by the glucometer check to be in the range of 3.9 to 7.9 mM, FTIR and QCL tests are conducted, to form a set of 41 concentration variables for each sensor configurations. For each concentration variable in this set of 41 variables, 3 measurements are taken with each sensor configuration, with 3 different sensor configurations. Hence, there are 4 sets of 123 noninvasive tests for these four patient-subjects.

For those taken with the QCL sensor equipped with a pressure actuator, all with nominal contact pressure of 20N/cm^2, the method of combs-to-spectrum translation is adopted. Thus, 4 patient-sets of 41 spectra are generated. One set is summarized in Figure 6 of the main text, and three sets are summarized in Figures S1a-c.

For those taken with the QCL sensor having no pressure actuator, the method of combs-to-spectrum translation is first used to generate 4 patient-sets of 41 spectra. All 4 sets of spectra are summarized in Figures S2a-d. In addition, all the raw combs are subjected to the MFDFA method from which 4 sets of 41 singularity spectra are generated. One set is summarized in Figure 7 of the main text, and three sets are summarized in Figures S3a-c.

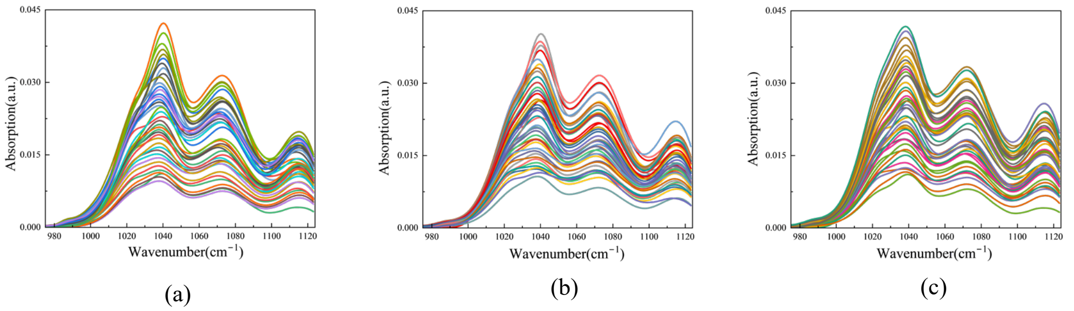

Figure S1. The photoabsorption spectra generated by the combs-to-spectrum-translation method for (a) Patient-Subject #2; (b) a Patient-Subject #3; (c) Patient-Subject #4, all tested with Sensor-System #1 with a pressure actuator set to a nominal contact-pressure of 20N/cm²; for each patient-subject, one spectrum is generated for one glucose concentration and a total of 41 spectra are shown.

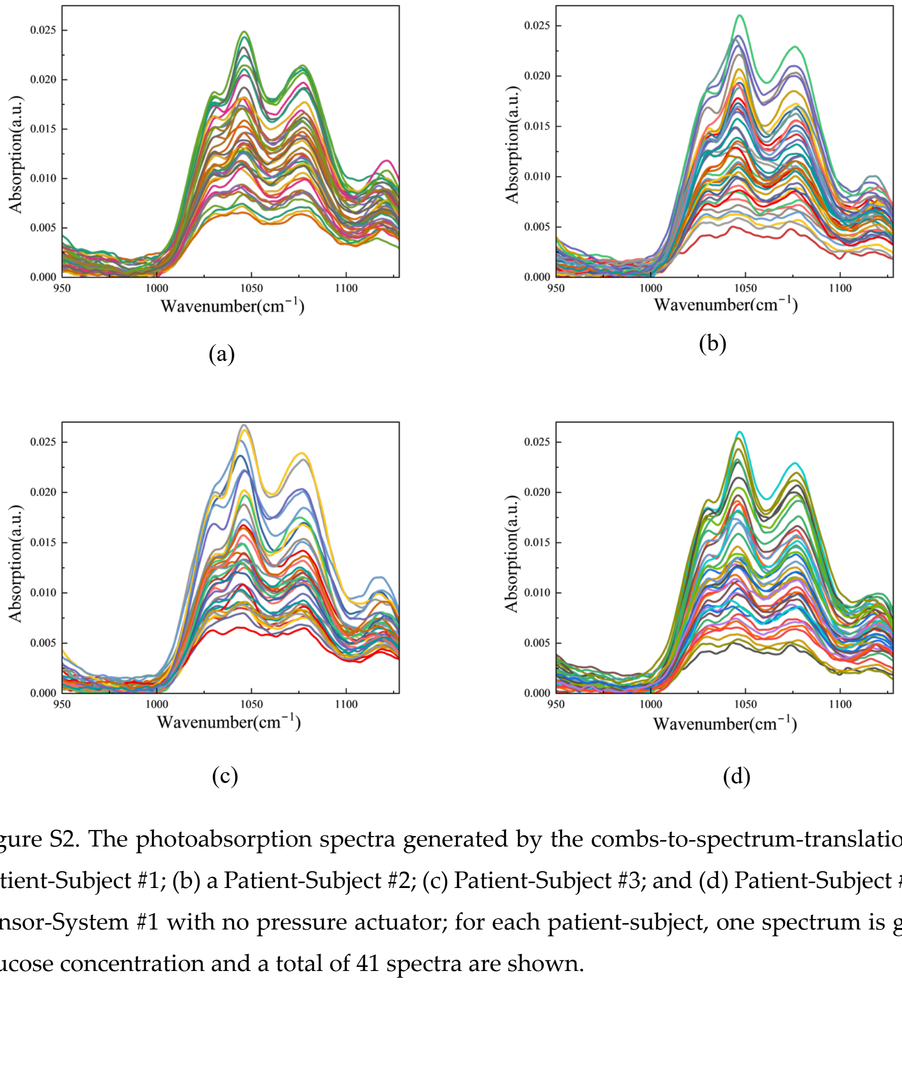

Figure S2. The photoabsorption spectra generated by the combs-to-spectrum-translation method for (a) Patient-Subject #1; (b) a Patient-Subject #2; (c) Patient-Subject #3; and (d) Patient-Subject #4, all tested with Sensor-System #1 with no pressure actuator; for each patient-subject, one spectrum is generated for one glucose concentration and a total of 41 spectra are shown.

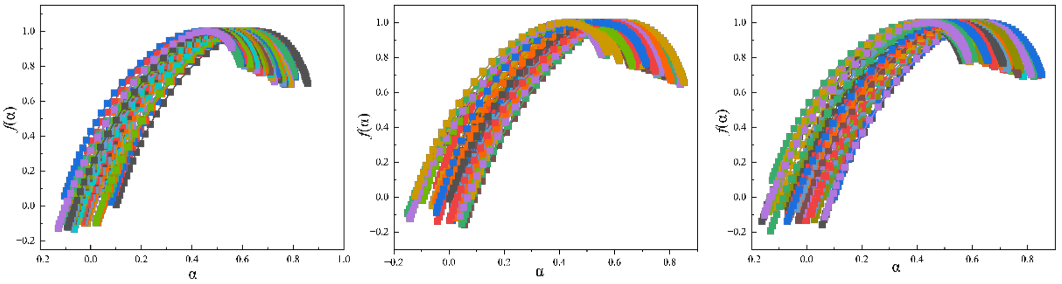

(a) (b) (c)

Figure S3. The singularity spectra directly deduced via MFDFA from the raw combs, with 41 singularity spectra for each patient-subject, (a) Patient-Subject #2; (b) a Patient-Subject #3; (c) Patient-Subject #4.

From the four singularity spectra, four sets of singularity strengths are extracted, and the data for Patient-Subject #1 are summarized in Table S2. The trends of these data are plotted in Figure S4. Evidently, each of the three primary singularity strengths, $\alpha_{+\infty}$, α_0, and $\alpha_{-\infty}$, correlates well with glucose concentration. The secondary singularity strength, $\Delta\alpha$, only weakly correlates to glucose concentration. All correlations are not linear but complex in nature; hence, the correlations are data mined with SVM.

Finally, the accuracy performance of Sensor-System #1 with no pressure actuator, Sensor-System #1 with pressure actuator, Sensor-System #2 with no pressure actuator, with data taken from Patient-Subjects #2-4 are summarized in Figure S5-7. Those for Patient-Subject #1 and the interpretations of the performance assessments are included in the main text.

Table S2. Singularity strengths of Patient-Subject #1

Number	Concentration	Singularity strengths			
		$\alpha_{+\infty}$	α_0	$\alpha_{-\infty}$	$\Delta\alpha$
1	3.9	0.0729	0.6263	0.7875	0.7145
2	4.0	0.0636	0.5874	0.7821	0.7185
3	4.1	0.0518	0.6117	0.7856	0.7338
4	4.2	0.0500	0.5656	0.7714	0.7213
5	4.3	0.0407	0.5576	0.768	0.7273
6	4.4	0.0689	0.6188	0.8204	0.7515
7	4.5	0.0754	0.641	0.8234	0.7479
8	4.6	0.0943	0.6253	0.8437	0.7494
9	4.7	0.0343	0.5351	0.7257	0.6914
10	4.8	0.0113	0.5655	0.726	0.7147
11	4.9	-7E-4	0.5371	0.7043	0.705
12	5.0	0.0554	0.621	0.8034	0.7479
13	5.1	-2E-4	0.5628	0.7293	0.7296
14	5.2	-0.0023	0.5511	0.7122	0.7145
15	5.3	-0.0082	0.5517	0.7256	0.7338
16	5.4	-0.0153	0.5478	0.7143	0.7296
17	5.5	-0.0243	0.5354	0.683	0.7074
18	5.6	-0.0187	0.5308	0.7069	0.7256
19	5.7	-0.0435	0.4972	0.6756	0.7191
20	5.8	-0.0935	0.444	0.6056	0.6991
21	5.9	-0.0243	0.5354	0.683	0.7074
22	6.0	-0.0807	0.4571	0.6243	0.705
23	6.1	-0.0421	0.5359	0.6845	0.7266
24	6.2	-0.0354	0.5502	0.7094	0.7447
25	6.3	-0.0359	0.5114	0.6664	0.7023
26	6.4	-0.1283	0.4476	0.5884	0.7168
27	6.5	-0.0398	0.5382	0.6868	0.7266
28	6.6	-0.1018	0.4403	0.5954	0.6972
29	6.7	-0.0421	0.5359	0.6845	0.7266
30	6.8	-0.0948	0.4683	0.5973	0.6921
31	6.9	-0.0631	0.5224	0.6693	0.7323
32	7.0	-0.1195	0.4503	0.5853	0.7048
33	7.1	-0.0508	0.5156	0.657	0.7078
34	7.2	-0.0518	0.5115	0.6519	0.7036
35	7.3	-0.1018	0.4403	0.5954	0.6972
36	7.4	-0.0521	0.5092	0.6549	0.707
37	7.5	-0.1136	0.4574	0.5921	0.7057
38	7.6	-0.0592	0.5141	0.6571	0.7164
39	7.7	-0.0631	0.5224	0.6693	0.7323
40	7.8	-0.0844	0.4984	0.6434	0.7278
41	7.9	-0.0947	0.4801	0.6261	0.7209

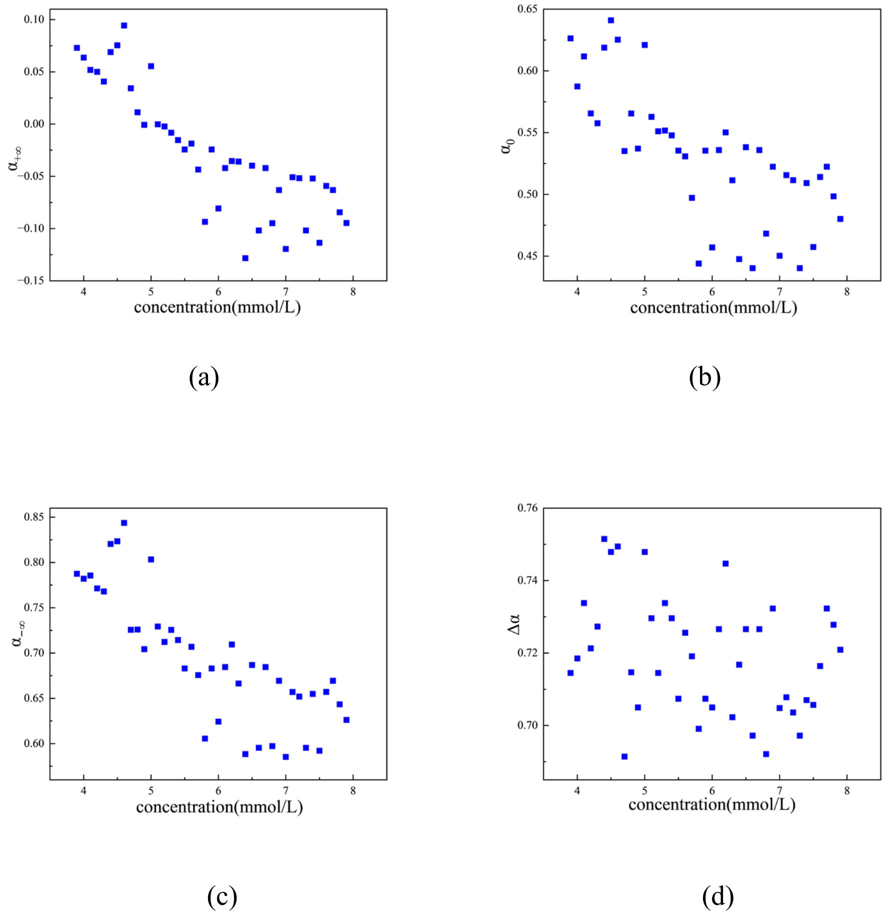

Figure S4. The correlation plots of singularity strengths vs glucose concentrations: (a) $\alpha_{+\infty}$,(b) α_0, (c) $\alpha_{-\infty}$, (d) $\Delta\alpha$ for Patient-Subject #1

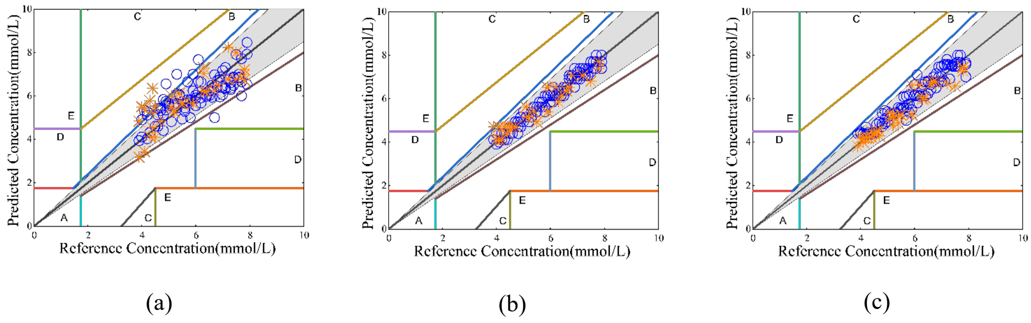

Figure S5 Clarke error grid plots corresponding to (a) Sensor-System #1 without pressure actuator; (b) Sensor-System #1 with pressure actuator; (c) Sensor-System #2 without pressure actuator for Patient-Subject #2. (The blue circles are data of the training set, and the orange crosses are data of the testing set.)

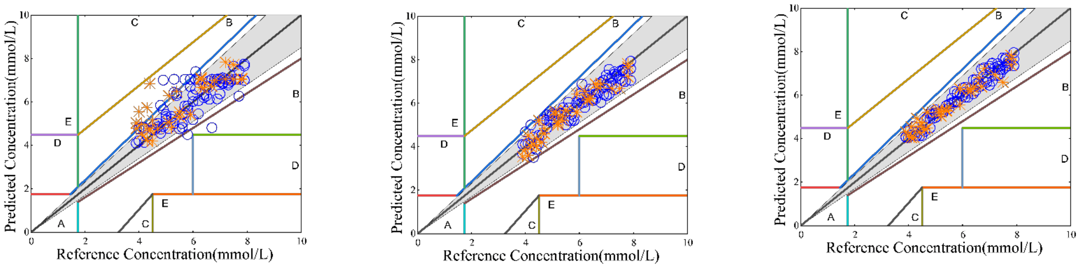

(a) (b) (c)

Figure S6 Clarke error grid plots corresponding to (a) Sensor-System #1 without pressure actuator; (b) Sensor-System #1 with pressure actuator; (c) Sensor-System #2 without pressure actuator for Patient-Subject #3. (The blue circles are data of the training set, and the orange crosses are data of the testing set.)

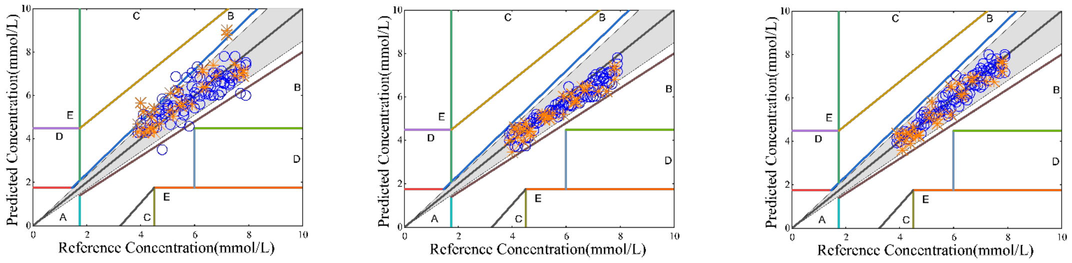

(a) (b) (c)

Figure S7 Clarke error grid plots corresponding to (a) Sensor-System #1 without pressure actuator; (b) Sensor-System #1 with pressure actuator; (c) Sensor-System #2 without pressure actuator for Patient-Subject #4. (The blue circles are data of the training set, and the orange crosses are data of the testing set.)

Reference

1. Kantelhardt, J.W., Zschiegner, S.A., Koscielny-Bunde, E., Havlin, S., Bunde, A.,Stanley, H.E., Multifractal detrended fluctuation analysis of nonstationary time series. *Phys. A* **2002,** *316,* pp. 87-114.
